# Weight reduction and cardiovascular benefits: Protocol for a systematic review and meta-analysis

**DOI:** 10.1097/MD.0000000000013246

**Published:** 2018-12-14

**Authors:** Yingke Zhao, Branda Yee-Man Yu, Yanfei Liu, Tiejun Tong, Yue Liu

**Affiliations:** a School of Chinese Medicine, Li Ka Shing Faculty of Medicine, The University of Hong Kong, Pokfulam, Hong Kong; b Cardiovascular diseases center, Xiyuan Hospital, China Academy of Chinese Medical Sciences, Beijing, China; c School of Nursing, the Hong Kong Polytechnic University, Kowloon, Hong Kong; d Graduate school of Beijing University of Chinese Medicine, Beijing, China; e Department of Mathematics, Hong Kong Baptist University, Kowloon Tong, Hong Kong.

**Keywords:** cardiovascular outcomes, obesity, systematic review, weight loss

## Abstract

**Background:**

There is widespread obesity paradox in cardiovascular diseases, the cardiovascular influence from weight management remains controversial. Moreover, previous publications indicating that different weight reduction extent might lead to various results. Thus, it is of importance to reassess the cardiovascular benefits of weight management strategies.

**Objectives:**

This review is designed to assess the association between weight loss and cardiovascular outcomes.

**Methods:**

Clinical trials including randomized control trials, observational studies reported a weight change before and after weight interventions including lifestyle intervention, as well as pharmacotherapies were included. Three major databases will be searched to retrieve the appropriate studies. Dual selection and abstraction of data will be conducted by 2 authors independently. The population, intervention, comparator, outcomes, study characteristics framework will be used to extract all the necessary data from included studies. The risk of bias assessment will be conducted in duplicate based on the Cochrane risk of bias guideline for randomized controlled trials (RCTs) and the Strengthening the Reporting of Observational Studies in Epidemiology (STROBE) statement for observational studies respectively. The primary outcomes will be the cardiovascular mortality, and the secondary outcomes are all-cause mortality and new cardiovascular events. A meta-analysis will be considered if there is sufficient homogeneity among selected studies. Follow the criteria of Grading of Recommendations, Assessment, Development and Evaluation (GRADE), the quality of the cumulative evidence will be evaluated.

**Results and conclusions:**

The results of this systematic review could provide reliable and concrete evidence for weight loss and its cardiovascular benefits.

Prospero registration number: CRD42018108582.

## 1 Introduction

Obesity is a surging problem in most countries listed as the second leading cause of preventable death following cigarette smoking.^[1]^ In the US, the prevalence of obesity (defined as body mass index [BMI]≥30) has almost reached 40% among citizen in 2016.^[2]^ It is widely accepted that obesity is closely associated with the increased risk of adverse health outcomes, such as elevated blood pressure, aggravated dyslipidemia, increased blood glucose, ultimately leads to a variety of diseases, such as hypertension, coronary heart disease, diabetes.^[3]^ According to the epidemiological study, cardiovascular diseases (CVDs) were the leading causes of death among obese, accounted for 2.7 million death, as overweight might attributed to cardiac ventricular structural and functional abnormalities.^[4]^ Furthermore, individuals who are obese or overweight tend to be more likely to develop CVDs, or unfavorable cardiovascular outcomes, showing a higher all-cause mortality.^[5]^

Nevertheless, evidence from large populational based-observational studies, systematic reviews suggested the widespread existence of obesity paradox, that the obese individuals tend to have a better prognosis than their lean counterparts.^[3,6–9]^ It is undoubted that deliberate weight management could improve the abnormal cardiac output, left ventricular hypertrophy, reverse the cardiac performance, and morphology in obese.^[10]^ Weight control could lead to diverse benefits, including decreased blood pressure, inflammatory cytokines, as well as improved lipid profiles.^[11–13]^ Moreover, the management of obesity, when accompanied by physical activity, exercise training, and fitness training, has been recommended by major guidelines as primary prevention for most CVDs, as well as diabetes.^[1,14]^

While Observational studies assessed the effects of intentional weight loss in patients with existing CVDs have reached diverse conclusions, ranging from modest benefits to harm.^[15,16]^ The large randomized controlled trials (RCTs), the Look AHEAD Study, which focusing on the intensive lifestyle-based weight control in patients with type 2 diabetes, showed numerous health benefits, but no significant effect on CVD survival.^[17,18]^ On the contrary, other 2 large population-based weight control study for diabetes, the Da Qing Diabetes prevention study, and PREDMIMED study observed significantly reduced in CVD incidence.^[19,20]^ Therefore, it is of importance to reassess the existing evidence related to weight loss and its cardiovascular implications. Recently, a systematic review comprehensively reviewed all the RCTs on dietary intervention for all-cause mortality, cardiovascular mortality, and cancer mortality, demonstrated that high quality evidence supported the benefits of dietary-guided weight reduction on the reduction of all-cause mortality (risk ratio 0.82, 95% confidence interval 0.71 to 0.95), though unfavorable effect on cardiovascular mortality (risk ratio 0.93, 95% confidence interval 0.67 to 1.31) or newly cardiovascular events (risk ratio 0.93, 95% confidence interval 0.83 to 1.04).^[21]^ It is noteworthy to point out that only the RCTs were included, and only the dietary-induced weight loss effect was assessed, and the weight loss magnitude was not reported, unfortunately, as it might also affect the cardiovascular survival.^[22]^ Thus, it is necessary to determine the ideal weight reduction extent for diverse participants, as well as the effects of different weight loss strategies, to provide a more practical and reliable weight management recommendation for patients, particularly these with cardiovascular concerns.

## 2 Objectives

The primary objective of this review is to investigate whether intentional weight loss was associated with satisfactory cardiovascular outcomes. In subgroup analyses, we will assess the association of weight reduction range among population subgroups, for example, stratified by age and existing diseases, and the different weight reduction modes, such as physical exercise, dietary supplementation and restriction, and therapeutic intervention.

## 3 Methods

### 3.1 Standards

The protocol was developed in accordance with the Preferred Reporting Items for Systematic Reviews and Meta-Analysis (PRISMA) statements and the guidelines in Cochrane Handbook for systematic Reviews of interventions.^[23]^

### 3.2 Protocol and registration

This systematic review protocol has been registered with the PROSPERO International Prospective Register of Systematic Reviews (CRD42018108582). A PRISMA-P checklist is attached (supplementary file)

### 3.3 Eligibility criteria

Publications meet the following criteria will be eligible:

(1) clinical trials including randomized control trials, controlled before and after studies, as well as cohort, case-control and cross-sectional studies;
(2) adults ≥ 18 years of age, provide “pre-post treatment” data linking weight differences;
(3) provide a measure of weight change before and after the specific interventions including lifestyle changes such as diet, exercise, cognitive behavioral therapy and/or therapeutic interventions (these included anti-obesity pharmacotherapy) were included. Interventions, as bariatric surgery were excluded because the procedures are invasive which cannot be generalized as a primary preventive treatment;
(4) reported data on cardiovascular outcomes;
(5) published in English up to 31 August 2018.

### 3.4 Outcome measurement

A comparison will be made with weight loss interventions versus no intervention. The primary outcome is cardiovascular mortality, and the secondary outcomes are all-cause mortality and cardiovascular events, including incidence of acute myocardial infarction (AMI), stroke, hospitalization for acute coronary syndrome and urgent revascularization procedures. The definition of cardiovascular mortality and cardiovascular events were according to the related statement in the American College of Cardiology/American Heart Association guidelines.^[24]^

### 3.5 Search methods

The following databases will be searched, the Cochrane Central Register of Controlled trials, the Cochrane Database of systematic review, Web of Science and PubMed of English-language publications. Cross-referencing from retrieved studies will be conducted additionally.

### 3.6 Search strategy

We will systematically search, and reference lists, using terms relating to the population, interventions, and outcomes. Table 1 demonstrates the proposed search terms. Only literature published between January 1990 and the date the research is complete in English will be retrieved. A preliminary search strategy for PubMed is demonstrated in Table 2. Search term will be adapted to other databases based on the specific requirements for each database. We also manually checked reference lists to identify other potential studies.

**Table 1:**
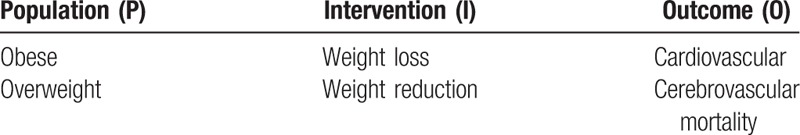
Key terms used for developing a comprehensive search strategy.

**Table 2:**
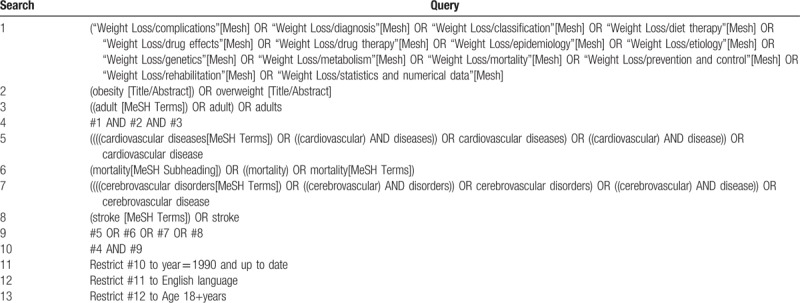
Preliminary search strategy: PubMed format.

### 3.7 Data management

All the literature search results will be combined and uploaded to one single EndNote (V.9) library. Duplicates will be removed.

### 3.8 Selection process

**Figure 1:**
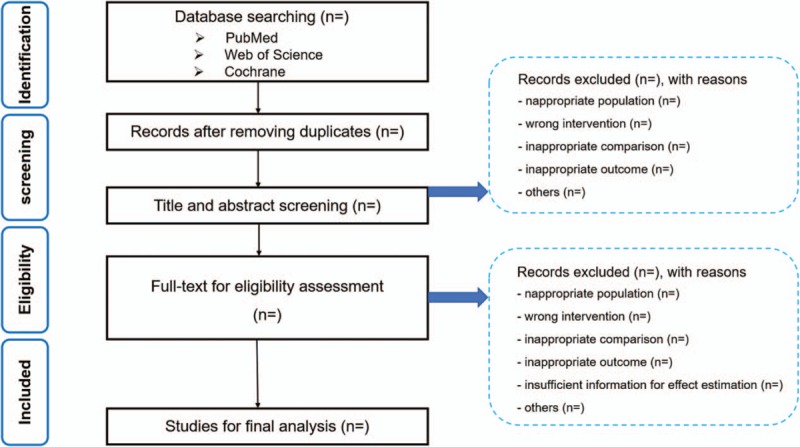
Flow chart of literature screening process.

The study selection will be accomplished via 2 stage. First, all the titles and abstracts will be screened by 2 researchers independently, to obtain which appear to meet the inclusion criteria or there is any uncertainty. Any discrepancies will be resolved by discussion. Next, both authors will then obtain the full-text articles to further identified these meet the inclusion criteria. Reasons for exclusion of articles in the full-text screening session will be documented as follows, inappropriate population, wrong intervention, inappropriate comparison, inappropriate outcome, insufficient information for effect estimation, others. Discrepancies will be discussed by 2 authors. Further consultation with a third review will be carried out if consensus cannot be reached. A proposed flow chart shown in Figure 1, illustrates the whole search process.

### 3.9 Data extraction

All the required data will be double extracted by 2 authors using a standardized data extraction form. Data referring to the study (author, journal, year, etc.), study settings, baseline characteristics of participants (age, sex, CV disease, BMI), intervention details for weight loss, weight measure methodology, outcome of interests, outcome measurement, length of follow-up period will be extracted. On completion, both authors will check the extraction data and resolve discrepancies. Authors will be contacted in the event of missing information. Before reach agreements, the overall agreement rate will be calculated using Cohen's κ statistic.

Moreover, the information for covariates measured and adjusted for will also be documented. Results for any additional stratified analyses will be further extracted when necessary. If stratified effects of interest are not reported or insufficient for further estimation, related information will be sought from the authors.

### 3.10 Risk of bias assessment

Risk of bias for RCTs will be assessed following the Cochrane risk of bias guideline.^[25]^ Observational studies will be assessed using the Strengthening the Reporting of Observational Studies in Epidemiology (STROBE) statement.^[26,27]^ Bias in RCTs will be assessed based on the following items: random sequence generation, allocation concealment, blinding, completeness outcome, and selective reporting. Each domain will be classified as “high risk”, “low risk”, and “unclear”. The overall risk will be defined as high if any one of the items is considered as high risk. Observational publications will be evaluated according to items introduced in the STROBE statements.^[26]^ The 2 authors will conduct the risk of bias assessment independently. Upon completion, they will then review the results of assessment. Disagreements will be solved first by discussion and then through consultation with a third expert. Summary risk of bias table will be produced.

### 3.11 Confidence in cumulative evidence

The strength of evidence will be assessed by the “Grades of Recommendation, Assessment, Development, and Evaluation (GRADE)” approach for the relating domains, such as the methodological flaws within the studies, the consistency of results across the studies.^[28]^ The quality of evidence will be ranked as high (future evidence is unlikely to change the conclusion obtained from our research), moderate (further studies might alter our conclusion), low (further evidence is needed to answer the involved research question with increased confidence).

### 3.12 Data synthesis

We will firstly adopt a narrative method for data synthesis; in which studies are grouped by targeted participants (with a history such as diabetes, CVDs, chronic cancers) to generally summarize the evidence for the relationship of weight loss and primary outcomes. In cases reporting multiple time points, the longest follow-up time point will be adopted ≤1 year.

If we find selected studies bearing sufficiently homogenous, our narrative synthesis will also described subgroup analyses, relevant to our secondary research question, including

(1) the effect of different weight interventions;
(2) the effect of weight loss on the secondary outcomes;
(3) the effect of weight loss for population characteristics, such as age strata and other common vascular risk factor;
(4) the association of weight loss extent and primary outcomes.

If there is sufficient evidence, we will consider conducting a meta-analysis to estimate the pooled effect. We will use the I^2^ statistic to assess the statistical heterogeneity and determine the accordingly model for further analysis (fixed or random effects model). All analyses will be conducted using the log of odds ratios (OR), then transformed back to OR for presentation purposes. The sources of heterogeneity will be identified by removing publications at highest risk of bias. Additionally, the potential reporting bias will also be considered by using funnel plots. If these are not possible we will discuss possible sources of bias across studies. All of the statistical analyses will be performed via RevMan and STATA.

### 3.13 Ethics and dissemination

Ethical review is not required as this protocol is for a systematic review since there is no direct involvement of patients in the whole process. We will report our results comprehensively for peer-reviewed journal and present the primary findings at important conferences.

## 4 Discussion

Obesity or overweight has been long regarded as a risk factor for cardiovascular diseases. On the other hand, the obesity paradox, is widely observed in most of the patients with existing CVDs, indicating a better prognosis for the obese patients. Results from large RCTs focusing on cardiovascular implications of weight loss interventions are controversial.^[17,19]^ It is necessary to revisiting the role of weight loss and its cardiovascular benefits.

### 4.1 Strengths and limitations of this study

- This review benefits from a comprehensive search strategy, designed to retrieve extensively studies related to our research problems. We anticipate our study could provide comprehensive and concrete evidence for the weight management. Thus, we tried to extend previous work by including numerous new studies and broad the interventions not restricted to lifestyle or pharmaceutical interventions.
- The present study will mainly focus on the cardiovascular health, thus we will try our best to carry out comprehensively subgroup analyses to examine the influence of weight loss on every cardiovascular outcome.
- Common to most aggregate data meta-analysis, significant and unavoidable heterogeneity may exist. Considering this, the statistical analysis will be accomplished with the guidance of experienced statistician.
- The common limitation of data aggregation is the risk of ecological fallacy. To deduce more reasonable conclusion, the results will be interpreted by experienced cardiovascular expert to give a more reliable and applicable recommendation for public.

## Author contributions

Yingke, Zhao drafted the preliminary version of this protocol. Yingke, Zhao, Branda Yee-Man Yu, Yanfei Liu will contribute to the literature search, screening, data selection, extraction, risk of bias assessment. The final analysis of data for all the included studies will be completed by Yingke, Zhao, with Tiejun Tong for statistical consultation, and Yue, Liu for cardiovascular related content. Yue, Liu, as corresponding author, is the guarantor of this review. All the 5 authors read and approved the final manuscript.

**Conceptualization:** Yue Liu.

**Data curation:** Yanfei Liu.

**Methodology:** Yingke Zhao, Branda Yee Man Yu, Yanfei Liu, Tiejun Tong.

**Project administration:** Yue Liu.

**Supervision:** Yue Liu.

**Writing – original draft:** Yingke Zhao.

**Writing – review & editing:** Branda Yee Man Yu, Tiejun Tong, Yue Liu.

Yue Liu orcid: 0000-0002-0084-863X.
